# Recurrent Self-Induced Nontraumatic Orbital Emphysema Causing Orbital Compartment Syndrome with Optic Nerve Dysfunction

**DOI:** 10.1155/2021/8884009

**Published:** 2021-03-11

**Authors:** S. Cutting, C. Davies-Husband, C. Poitelea

**Affiliations:** ^1^East Kent University Hospitals NHS Trust, UK; ^2^Brighton and Sussex University Hospitals NHS Trust, UK; ^3^Western Sussex Hospitals NHS Trust, UK

## Abstract

The majority of cases of orbital emphysema are due to trauma. Complications are rare, and therefore, the need for surgical intervention is uncommon. We present the first case of which we are aware in which nontraumatic orbital emphysema led to orbital compartment syndrome and subsequent optic nerve dysfunction. The patient underwent emergency needle decompression. A 51-year-old man presented to the Emergency Department with right-sided unilateral proptosis, reduced visual acuity, and binocular diplopia. This occurred after performing a Valsalva manoeuvre with no history of head trauma. He also mentioned that over the past year he had experienced multiple episodes of transient proptosis occurring after Valsalva manoeuvres. Visual acuity in the right eye was reduced to 6/21. A relative afferent pupillary defect was present and intraocular pressure (IOP) was 12 mmHg. The CT scan showed significant orbital emphysema in the medial aspect of the right orbit. Needle decompression was performed resulting in immediate resolution of his symptoms. This case demonstrates that, in cases of orbital emphysema, a lack of a history of trauma and a normal IOP cannot always be used to rule out serious pathology.

## 1. Introduction

The majority of cases of orbital emphysema are due to trauma causing orbital wall fractures [1, 2]. Signs and symptoms suggestive of orbital emphysema are enophthalmos or proptosis, crepitus, diplopia, and protrusion on nose-blowing [1, 3]. Complications are uncommon and so is the need for intervention, but if severe enough to cause orbital compartment syndrome, it can have disastrous effects on vision through central retinal artery occlusion (CRAO) or compressive optic neuropathy [2, 4]. The acute presentation of orbital emphysema may not occur immediately after trauma. Instead, it may present days or weeks later after a Valsalva manoeuvre increases intranasal pressure. This can cause air to become trapped in the orbit via a one-way valve mechanism where it is likely that structures such as orbital fat prevent air leaving the orbit through the bony defect. Smaller fractures may be particularly prone to this phenomenon [2]. There have been case reports of rare, nontraumatic causes of orbital emphysema. These include surgery, infection, compressed air, osteomas or pneumocoeles of the paranasal sinuses and barotrauma [5]. Orbital emphysema due to pressure changes during air travel in a young man with recent traumatic orbital floor fracture has also been described [6]. Rarely, Valsalva manoeuvres have resulted in orbital emphysema without a history of prior trauma. Orbital wall fractures have been identified in a proportion of these cases, thought to be a result of the rise in intranasal pressure sneezing or nose-blowing confers [5]. Studies have quantified elevated intranasal pressures of 8 mmHg in the former but strikingly in excess of 70 mmHg in the latter [7, 8]. Cases of nontraumatic orbital emphysema tend to be mild and can be managed conservatively. We present an unusual case with recurrent self-induced, nontraumatic orbital emphysema which ultimately resulted in orbital compartment syndrome requiring urgent decompression.

## 2. Case Presentation

A 51-year-old man presented to the Emergency Department late at night complaining of proptosis of his right eye with accompanying diplopia. He provided a history of attempting to “unblock his ears” by performing a Valsalva manoeuvre. He stated he performed this Valsalva manoeuvre habitually on a daily basis, which often resulted in proptosis of the right eye that he was able to manually push back into place. He had not previously sought medical advice for this. His past medical history included Meniere's disease for which he performs Valsalva manoeuvres in an attempt to relieve his symptoms. His remaining past medical history was a radical mastoidectomy 22 years previously for cholesteatoma. There was no prior history of trauma.

Examination showed nonaxial proptosis of the right eye with infero-lateral displacement of the globe. Visual acuity in this eye was reduced to 6/21 (compared to 6/12 in the left eye). Binocular diplopia was apparent. He retained a good range of eye movement, and visual fields were normal to confrontation. Pupillary examination confirmed a relative afferent pupillary defect (RAPD); intraocular pressure (IOP) measure by Goldmann applanation was within the normal range at 12 mmHg, and fundoscopy demonstrated no optic nerve changes.

**Figure 1:**
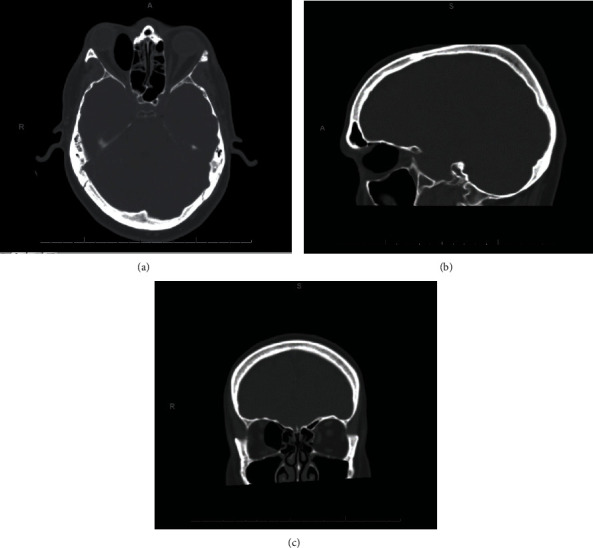
CT scan of patient with (a) axial, (b) sagittal, and (c) coronal views showing a 36 × 26 × 16 mm collection of gas in the medial aspect of right orbit causing displacement of intraorbital structures and resultant.

Accordingly, an urgent CT of the orbit was arranged, which showed significant orbital emphysema in the medial aspect of the orbit (Figure 1). There was a suggestion of a small bony defect in the superior aspect of the medial orbital wall. After a multidisciplinary discussion jointly between ophthalmology and otolaryngology consultants, preparations were provisionally made for urgent surgical decompression via endoscopic approach. However, on detailed review of the cross-sectional images, needle decompression was proposed in the first instance.

Sterile preparation was administered using aqueous chlorhexidine, followed by 2 ml lignospan with 1/80,000 adrenaline infiltration into the right conjunctival mucosa. Using a syringe attached to a 16G cannula inserted adjacent to the carcuncle, 7 ml of air was subsequently aspirated from the orbit resulting in immediate resolution of his proptosis and diplopia, with concurrent improvement in visual acuity. IOP after the procedure was 17 mmHg in the right eye. As both pupils had been dilated to perform fundoscopy, assessment for resolution of the RAPD was not possible. A prophylactic dose of 375 mg coamoxiclav TDS was provided for five days. Ophthalmology follow-up was arranged for the next day, and the patient provided with information to avoid further nose-blowing/Valsalva manoeuvres, with strict instruction to return to casualty urgently if he experienced deterioration in vision. The patient subsequently declined follow-up and stated by telephone that this was not required due to lasting resolution in proptosis and diplopia, with concurrent normal visual acuity.

## 3. Discussion

Most reported cases of orbital emphysema follow a history of trauma, though not necessarily immediately [1, 2]. Patients with a preceding episode of trauma days or weeks before may present acutely with orbital emphysema after an episode of increased intranasal pressure provoked by sneezing or nose-blowing. However, in this case, there was no preceding injury noted. From a review of the literature, we have identified only 21 cases of orbital emphysema where no history of trauma or periocular surgery was evident [8–28]. Cases of orbital emphysema are usually mild and do not affect visual function, with approximately 12% of patients requiring decompression [5]. Of the 21 cases found with no history of trauma [8–28], one had nonurgent surgery due to persistent diplopia lasting more than a week [17]. In this case, fractures of the orbital floor were apparent, with fat entrapment. Two of the 21 cases reported emergency needle decompression [9, 12]. In the first, there was proptosis, restriction of eye movements, raised intraocular pressure (IOP), and a hazy cornea. However, it was noted that there was no RAPD or dyschromatopsia and therefore no evidence of optic nerve compromise. In the second, decompression was performed due to the extent of eyelid swelling precluding assessment of the eye. As such, our case is the first to document clear evidence of optic nerve dysfunction after nontraumatic orbital emphysema.

In addition, recurrent orbital emphysema is relatively rare and in our case contributed to by the patient's lack of desire to seek medical attention. Two posttraumatic cases describe short-term recurrence of symptoms due to increases in intranasal pressure: Valsalva manoeuvres while crying in one case and sneezing in the other [5, 29]. In our case, while the patient was not trying to cause proptosis of his eye, he was aware this was a consequence of him performing Valsalva manoeuvres. However, this practice was continued for a year and only led him to seek medical attention once it had become so severe as to cause optic nerve dysfunction. Given that he performed Valsalva manoeuvres on a daily basis, it may be that he already had a small pocket of intraorbital air prior to this acute episode which may be responsible for the severe and acute nature of optic nerve compromise.

Finally, the IOP was 12 mmHg prior to decompression despite significant intraorbital air, proptosis, and RAPD. This emphasises the fact that a normal IOP cannot exclude orbital compartment syndrome. Intraocular pressure is often used as a surrogate for intraorbital pressure. Documented IOP in all previously reported cases of orbital emphysema (both traumatic and nontraumatic) requiring emergency decompression ranged from 20 to over 100 mmHg (Table 1) [2, 6, 9, 30–38]. As such, our case highlights that while raised IOP can act as a surrogate measure to inform the decision for urgent orbital decompression, a normal measurement does not exclude optic nerve compromise.

In summary, we present the first documented case in which nontraumatic orbital emphysema resulted directly in orbital compartment syndrome with optic nerve dysfunction. The authors stress that the absence of a history of trauma and a normal IOP cannot definitively exclude the necessity for urgent orbital decompression.

## Figures and Tables

**Table 1:** Intraocular pressure in cases of orbital emphysema in which patients underwent emergency decompression.

Reference	Cases which underwent emergency decompression	IOP prior to decompression
Fleishman et al. 1984 [30]	2	(i) “Over 100” (ii) Not stated
Dobler et al. 1993 [31]	1	45
Hunts et al. 1994 [2]	7	(i) 20 (ii) 37 (iii) 35 (iv) 28 (v) Not stated (vi) “Normal” (vii) 21
Wearne et al. 1998 [32]	1	20
Monaghan and Millar 2002 [6]	1	Not stated
Lee et al. 2006 [33]	1	80
Chaudry et al. 2007 [34]	1	25
Singh et al. 2007 [9]	1	37
Furlani et al. 2009 [35]	1	23^∗^
Chak et al. 2012 [36]	1	46
Rowh et al. 2015 [37]	1	79
Lin et al. 2016 [38]	1	29

^∗^Measured at 23 initially but only underwent emergency decompression when situation progressed after more sneezing (no remeasurement of IOP stated).

## Data Availability

No data were used to support this study.

## References

[1] Birrer R. B., Robinson T., Papachristos P. (1994). Orbital emphysema: how common, how significant?. *Annals of Emergency Medicine*.

[2] Hunts J. H., Patrinely J. R., Holds J. B., Anderson R. L. (1994). Orbital emphysema: staging and acute management. *Ophthalmology*.

[3] O'Malley C. C. (1943). Orbital emphysema simulating cellulitis. *The British Journal of Ophthalmology*.

[4] Zimmer-Galler I. E., Bartley G. B. (1994). Orbital emphysema: case reports and review of the literature. *Mayo Clinic Proceedings*.

[5] Roelofs K. A., Starks V., Yoon M. K. (2019). Orbital emphysema: a case report and comprehensive review of the literature. *Ophthalmic Plastic & Reconstructive Surgery*.

[6] Monaghan A. M., Millar B. G. (2002). Orbital emphysema during air travel: a case report. *Journal of Cranio-Maxillo-Facial Surgery*.

[7] Clement P., Chovanova H. (2003). Pressures generated during nose blowing in patients with nasal complaints and normal test subjects. *Rhinology*.

[8] Halpenny D., Corbally C., Torreggiani W. (2012). Blowout fracture of the orbital floor secondary to vigorous nose blowing. *Irish Medical Journal*.

[9] Singh M., Phua V. M., Sundar G. (2007). Sight-threatening orbital emphysema treated with needle decompression. *Clinical & Experimental Ophthalmology*.

[10] Mohan B., Singh K. P. (2001). Bilateral subcutaneous emphysema of the orbits following nose blowing. *The Journal of Laryngology and Otology*.

[11] Singhai S. K., Dass A., Singh G. B., Virk R. D. (2003). Orbital pneumatocele. *Indian Journal of Otolaryngology and Head & Neck Surgery*.

[12] García-Medina J. J., García-Medina M., Pinazo-Durán M. D. (2018). Severe orbitopalpebral emphysema after nose blowing requiring emergency decompression. *European Journal of Ophthalmology*.

[13] Chiu W. C., Huang T. Y., Ku W. C., Lih M., Wang W. (2008). Spontaneous orbital subcutaneous emphysema after sneezing. *The American Journal of Emergency Medicine*.

[14] Di Lella F., Bacciu A., Vincenti V., Pasanisi E., Bacciu S. (2008). Orbital and infratemporal fossa emphysema following nose blowing. *Clinical Otolaryngology*.

[15] Rosh A. J., Sharma R. (2008). Orbital emphysema after nose blowing. *The Journal of Emergency Medicine*.

[16] Garcia De Marcos J. A., del Castillo-Pardo de Vera J. L., Calderón-Polanco J. (2008). Orbital floor fracture and emphysema after nose blowing. *Oral and Maxillofacial Surgery*.

[17] Rahmel B. B., Scott C. R., Lynham A. J. (2010). Comminuted orbital blowout fracture after vigorous nose blowing that required repair. *The British Journal of Oral & Maxillofacial Surgery*.

[18] Khader Q. A., Abdul-Baqi K. J. (2010). Orbital emphysema after a protracted episode of sneezing in a patient with no history of trauma or sinus surgery. *Ear, Nose, & Throat Journal*.

[19] Sen D., Chaturvedi P. K. (2011). Orbital emphysema after sneezing: a case report. *Medical Journal, Armed Forces India*.

[20] Watanabe T., Kawano T., Kodama S., Suzuki M. (2019). Orbital blowout fracture caused by nose blowing. *Ear, Nose, & Throat Journal*.

[21] Pausch N. C., Neff A., Dhanuthai K., Sirintawat N., Vorakulpipat C., Pitak-Arnnop P. (2014). Grand rounds: eyelid swelling after nose blowing. *American Journal of Otolaryngology*.

[22] Ozdemir O. (2015). Orbital emphysema occurring during weight lifting. *Seminars in ophthalmology*.

[23] Jawaid M. S. (2015). Orbital emphysema: nose blowing leading to a blown orbit. *BMJ Case Reports*.

[24] Betances R. F., Chiesa E. C., Osorio V. A. (2016). Orbital emphysema after blowing the nose. *Acta Otorrinolaringológica Española*.

[25] Hu H. C., Chang A., Chiu Y. H. (2017). Orbital emphysema after nose blowing. *QJM*.

[26] Ali S., Ranney M. L., Jarman A. F. (2018). Transient orbital compartment syndrome caused by spontaneous lamina papyracea dehiscence. *R.I. Medical Journal*.

[27] Myers S., Bell D. (2018). Orbital blowout fracture from nose blowing. *BMJ Case Reports*.

[28] Aubin-Lemay C., Acar P., Alnaif N., Alamri A., Azzi A. J., Cugno S. (2018). Orbital subcutaneous emphysema in a child. *The Journal of Craniofacial Surgery*.

[29] Marzuillo P., Aliberti F., Tipo V. (2016). Pneumo-orbita mimicking hordeolum. *Archives of Disease in Childhood*.

[30] Fleishman J. A., Beck R. W., Hoffman R. O. (1984). Orbital emphysema as an ophthalmologic emergency. *Ophthalmology*.

[31] Dobler A. A., Nathenson A. L., Cameron J. D., Carpel E. T., Janda A. M., Pederson J. E. (1993). A case of orbital emphysema as an ocular emergency. *Retina*.

[32] Wearne M. J., Frank J., Bryan S. (1998). Management of orbital emphysema. *Eye (London, England)*.

[33] Lee S. L., Mills D. M., Meyer D. R., Silver S. M. (2006). Orbital emphysema. *Ophthalmology*.

[34] Chaudhry I. A., Al-Amri A., Shamsi F. A., Al-Rashed W. (2009). Visual recovery after evacuation of orbital emphysema. *Orbit*.

[35] Furlani A. B., Diniz B., Bitelli L. G., Martins E. N. (2009). Enfisema orbitário compressivo após asseio nasal: relato de caso. *Arquivos Brasileiros de Oftalmologia*.

[36] Chak G., Joseph J. M., Tao J. P. (2012). Needle decompression of acute orbital emphysema: case report with video. *The British Journal of Ophthalmology*.

[37] Rowh A. D., Ufberg J. W., Chan T. C., Vilke G. M., Harrigan R. A. (2015). Lateral canthotomy and cantholysis: emergency management of orbital compartment syndrome. *The Journal of Emergency Medicine*.

[38] Lin C. Y., Tsai C. C., Kao S. C., Kau H. C., Lee F. L. (2016). Needle decompression in a patient with vision-threatening orbital emphysema. *Taiwan Journal of Ophthalmology*.

